# Diagnostic and prognostic gene expression signatures in 177 soft tissue sarcomas: hypoxia-induced transcription profile signifies metastatic potential

**DOI:** 10.1186/1471-2164-8-73

**Published:** 2007-03-14

**Authors:** Princy Francis, Heidi Maria Namløs, Christoph Müller, Patrik Edén, Josefin Fernebro, Jeanne-Marie Berner, Bodil Bjerkehagen, Måns Åkerman, Pär-Ola Bendahl, Anna Isinger, Anders Rydholm, Ola Myklebost, Mef Nilbert

**Affiliations:** 1 Department of Oncology, Institute of Clinical Sciences, Lund University, Lund, Sweden; 2 Department of Tumor Biology, Rikshospitalet – Radiumhospitalet Health Centre, Oslo, Norway; 3 Department of Theoretical Physics, Lund University, Lund, Sweden; 4 Department of Pathology, Rikshospitalet – Radiumhospitalet Health Centre, Oslo, Norway; 5 Department of Pathology, Institute of Clinical Sciences, Lund University, Lund, Sweden; 6 Department of Orthopedics, Institute of Clinical Sciences, Lund University, Lund, Sweden; 7 Department of Molecular Bioscience, University of Oslo, Norway

## Abstract

**Background:**

Soft tissue sarcoma (STS) diagnosis is challenging because of a multitude of histopathological subtypes, different genetic characteristics, and frequent intratumoral pleomorphism. One-third of STS metastasize and current risk-stratification is suboptimal, therefore, novel diagnostic and prognostic markers would be clinically valuable. We assessed the diagnostic and prognostic value of array-based gene expression profiles using 27 k cDNA microarrays in 177, mainly high-grade, STS of 13 histopathological subtypes.

**Results:**

Unsupervised analysis resulted in two major clusters – one mainly containing STS characterized by type-specific genetic alterations and the other with a predominance of genetically complex and pleomorphic STS. Synovial sarcomas, myxoid/round-cell liposarcomas, and gastrointestinal stromal tumors clustered tightly within the former cluster and discriminatory signatures for these were characterized by developmental genes from the EGFR, FGFR, Wnt, Notch, Hedgehog, RAR and KIT signaling pathways. The more pleomorphic STS subtypes, e.g. leiomyosarcoma, malignant fibrous histiocytoma/undifferentiated pleomorphic sarcoma and dedifferentiated/pleomorphic liposarcoma, were part of the latter cluster and were characterized by relatively heterogeneous profiles, although subclusters herein were identified. A prognostic signature partly characterized by hypoxia-related genes was identified among 89 genetically complex pleomorphic primary STS and could, in a multivariate analysis including established prognostic markers, independently predict the risk of metastasis with a hazard ratio of 2.2 (*P *= 0.04).

**Conclusion:**

Diagnostic gene expression profiles linking signaling pathways to the different STS subtypes were demonstrated and a hypoxia-induced metastatic profile was identified in the pleomorphic, high-grade STS. These findings verify diagnostic utility and application of expression data for improved selection of high-risk STS patients.

## Background

Soft tissue sarcomas (STS) account for ~1% of all malignancies and represent a heterogeneous group of mesenchymal tumors, the clinical management of which is challenging and requires multidisciplinary efforts that take the combined information from clinical investigations, imaging, histopathology, and cytogenetic and molecular genetic analyses into account. STS comprise more than 30 histological subtypes, although malignant fibrous histiocytoma (MFH)/undifferentiated pleomorphic sarcoma (UPS), liposarcoma, leiomyosarcoma, synovial sarcoma (SS), and malignant peripheral nerve sheath tumor (MPNST) account for three-fourth of the tumors [1]. Some STS subtypes are characterized by specific chromosomal translocations causing novel gene fusions e.g. *SS18-SSX *in SS and *TLS-CHOP *in myxoid/round-cell liposarcoma. However, a large group of STS, e.g. MFH/UPS, leiomyosarcoma, and dedifferentiated/pleomorphic liposarcoma, lack known specific recurrent alterations and are characterized by a multitude of rearrangements, amplifications, deletions, and somatic alterations, including mutations in *TP53*, deletions of *RB1 *and *CDKN2A*, and amplifications of *MDM2 *and *CDK4 *[2,3].

Gene expression studies have revealed diagnostic profiles and upregulation of specific pathways in sarcomas with type-specific genetic defects, e.g. SS, dermatofibrosarcoma protuberans, clear-cell sarcoma, Ewing sarcoma, rhabdomyosarcoma and gastrointestinal stromal tumors (GIST) [4-14] and have provided potential targets for novel therapies [15-18]. From a diagnostic point of view, the above mentioned tumor types can be identified from their underlying gene fusions, but the expression data reveal novel genes and pathways including multiple downstream targets of the resultant chimeric transcription factors, which provide a basis for the understanding of key biological changes in STS development. In contrast, molecular classification of the predominant, genetically complex STS subgroups, e.g. MFH/UPS, leiomyosarcoma, and dedifferentiated/pleomorphic liposarcoma, has been difficult with extensive pleomorphism that has precluded identification of recurrent profiles [9,10,12,14,19,20]. STS are highly malignant and metastases develop unpredictably in one-third of the cases, therefore, new prognostic markers would be of great clinical value. However, there have been only two reports of expression profiles associated with poor outcome in STS, both in leiomyosarcomas [21,22]. With the aim to establish diagnostic expression profiles for STS and to assess whether gene expression profiling can provide prognostic information, we used 27 k cDNA microarrays to characterize the expression patterns in a mixed series of 177 STS, with particular focus on high-grade pleomorphic tumors.

## Results

### Diagnostic Signatures

#### Unsupervised analyses

**Figure 1:**
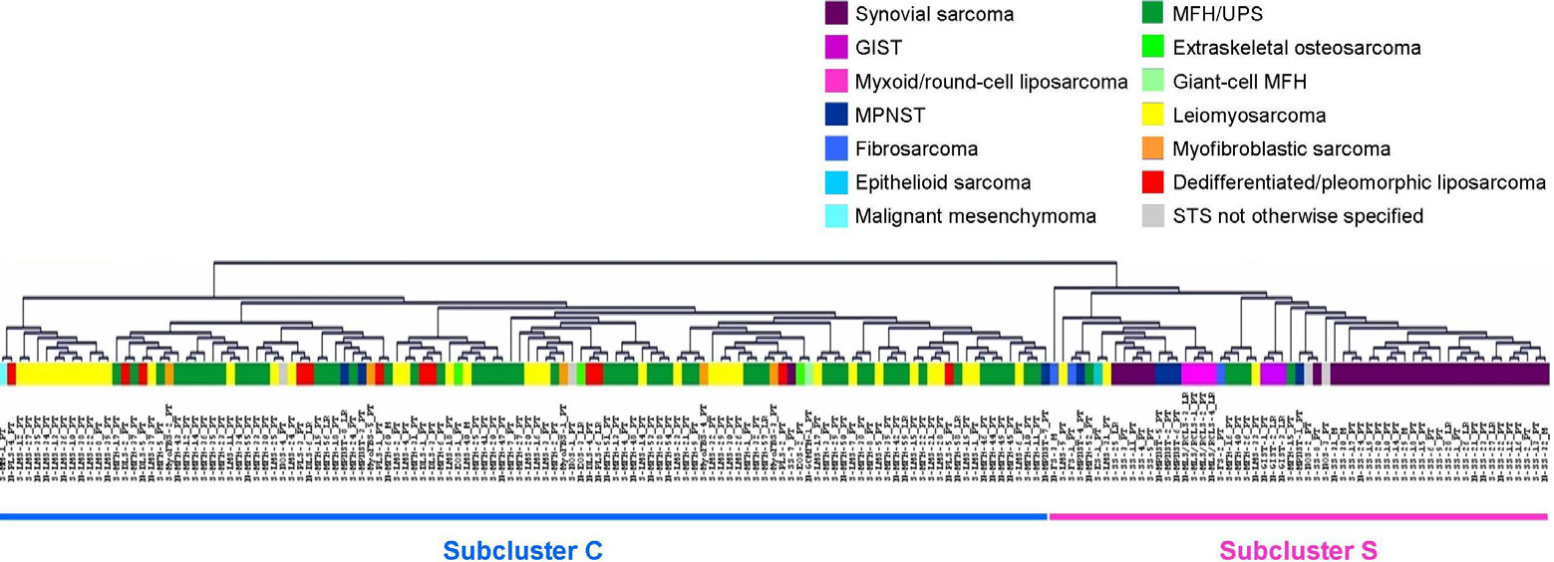
Unsupervised cluster analysis of the 177 STS samples resulted in two major subclusters: **C **dominated by pleomorphic STS subtypes with complex genetic alterations and **S **mainly containing STS of distinct histopathological subtypes with specific fusion genes or mutations.

When the 177 STS were subjected to unsupervised cluster analysis, based on the 6140 spots that passed the filter criteria, the dendrogram split into two major branches (Figure 1). One branch (referred to as S for simple/specific) consisted mainly of STS with simple, type-specific genetic defects and contained 31/32 SS, all 4 myxoid/round-cell liposarcomas, all 3 GIST and all 3 fibrosarcomas, in addition to 5/8 MPNST, 3/40 leiomyosarcomas, 5/60 MFH/UPS, 2/4 STS not otherwise specified (NOS) and the single epithelioid sarcoma. Herein, SS, GIST, myxoid/round-cell liposarcomas, and MPNST formed tight subclusters. The other branch (referred to as C for complex) consisted mainly of genetically complex, often pleomorphic STS subtypes and contained 55/60 MFH/UPS, 37/40 leiomyosarcomas, all dedifferentiated/pleomorphic liposarcomas, myofibroblastic sarcomas, and extraskeletal osteosarcomas along with the remaining 3 MPNST, 2 STS NOS, 1 SS and the single giant-cell MFH and malignant mesenchymoma. A subset of 11 leiomyosarcomas formed a distinct tight subcluster within C. When the 17 xenograft samples were included, a similar pattern of unsupervised clustering was observed and all 3 xenografts derived from tumors included in the study clustered next to their respective patient samples [see Additional file 1] suggesting that expression patterns of xenografts well reflect those of patient tumor material. A separate unsupervised cluster analysis of the 19 liposarcomas (16 tumors and 3 xenografts) separated the 5 myxoid/round-cell liposarcomas (characterized by the *TLS-CHOP *fusion) from the 14 dedifferentiated/pleomorphic liposarcomas (with complex genetic alterations) [see Additional file 2].

#### Supervised analyses and discriminatory gene lists

Discriminatory gene lists were generated by ranking genes based on their Golub-scores and performing 1000 random permutations to establish false-discovery rates (FDR) [see Additional files 3 and 4]. As expected, the strongest expression signals (with gene lists containing between 900 and 4000 genes with FDR of 11%) characterized STS subtypes with specific genetic defects, e.g. SS, GIST and myxoid/round-cell liposarcoma. The MFH/UPS, leiomyosarcoma, and MPNST were characterized by discriminatory gene expression signals containing 90 – 300 genes with FDR of 11%. Weak signals with high FDR characterized the fibrosarcoma (50% FDR for top 200) and dedifferentiated/pleomorphic liposarcoma (66% FDR for top 150), whereas myofibroblastic sarcoma and extraskeletal osteosarcoma were small heterogeneous groups without discriminatory profiles.

#### Synovial Sarcoma

The SS were characterized by the strongest expression signature containing 4000 differentially expressed genes with a FDR of only 11%. Multiple developmental pathways that interact to regulate embryonic development and organogenesis were upregulated in SS. These genes included *ERBB2*, *FGFR1*, *FGFR3*, *FGF18*, and *FRAG1 *from the EGF and FGF receptor signaling pathways, members of the Hedgehog (Hh) signaling pathway like *PTCH*, *SMO*, *BMP7*, *FOXM1 *and *CSNK1E*, retinoic acid receptor (RAR) pathway genes like *RARA*, *RARG*, *MDK*, *MEIS1 *and *PRAME*, and genes involved in Notch receptor signaling like *NOTCH1*, *JAG1 *and the transducin-like enhancer of split genes. EASE (Expression Analysis Systematic Explorer) identified overexpression of the Wnt receptor signaling pathway including *AXIN2*, *LEF1*,* TCF7*, *WISP2 *and the frizzled homologs, and the TGF-β signaling pathway including *RUNX3*, *SMAD6*, *TGFB1*, *TGFB2 *and the bone morphogenetic proteins. One of the largest functional groups of upregulated genes was that involved in chromatin remodeling including several histones and SWI/SNF related matrix associated actin dependent regulator of chromatin (SMARC) genes. A large number of neural differentiation genes like *EFNA1*, *NCAM1*, *NEDD5*, *NPDC1 *and *OLFM1*, ribosomal protein genes, many forkhead box transcription factors and the SS chromosome X breakpoint genes *SSX1 *and *SSX3 *were also highly expressed [see Additional files 5 and 6].

#### Gastrointestinal stromal tumor

The GIST, with activating mutations in the *KIT *gene were characterized by a distinct expression profile of 900 genes (11% FDR), including top overexpressed genes involved in the KIT receptor signaling pathway, e.g. *KIT*, and *PRKCQ *(*SCF*, *PIK3CB *and *PIK3R1 *were also overexpressed but a bit further down the list beyond the 11% FDR cut-off), and other developmental pathways, e.g. *BMP4*,  *FGF2*, *IGF2*, *SFRP1 *and *TLE4*, as well as neurogenesis and neural differentiation genes like *SMPD1*, *HOXA4*, *CIT*, *HOXA9*, *SIM2*, *NPTX1*, *NEDD5 *and *DCTN1*. Other groups of highly expressed genes identified in the EASE analysis were those involved in protein transport, lipid metabolism and kinase activity [see Additional files 7 and 8].

#### Myxoid/round-cell liposarcoma

A distinct expression signature of about 1000 differentially expressed genes (11% FDR) characterized the myxoid/round-cell liposarcomas. Herein, several lipid metabolism genes, including *DGKD*,* EBPL*, *FABP5*, *LPL*, *MGLL *and *PPARG *were upregulated, as were many developmental genes, ribosomal subunit genes and genes involved in amino acid and carboxylic acid metabolism [see Additional files 9 and 10].

#### Malignant peripheral nerve sheath tumor

Golub-score analysis identified a 100-gene signature (11% FDR) characterized by overexpression of developmental pathway genes like *FRAG1*, *WISP2*, *RARRES3*,* SPRY1 *and *SMO *from, e.g. the Wnt, RAR and Hh signaling pathways. Moreover, genes related to neural development such as *NEDD4*, *NPDC1*, *GSTP1*, and *DSCR1*, and several ribosomal protein genes were also highly expressed [see Additional files 11 and 12].

#### Leiomyosarcoma

A 200-gene signature (11% FDR) characterized by overexpression of muscle-specific genes like *ACTN3*, *CALD1*, *MBNL1*, *MLC1*,* MYH11*, *MYL4*, *SLMAP*, *TPM2 *and *TAGLN3 *was identified in the leiomyosarcomas. EASE analysis also identified upregulation of carbohydrate metabolism and energy pathway genes [see Additional files 13 and 14].

#### Malignant fibrous histiocytoma/undifferentiated pleomorphic sarcoma

A 300-gene signature (11% FDR) was identified for the MFH/UPS samples and the top upregulated functional groups included cathepsins and genes related to protein degradation, inflammatory response, cell motility, proliferation, cell-cycle control, and intracellular signaling [see Additional files 15 and 16].

#### Discriminatory gene lists with high FDR

The gene lists for dedifferentiated/pleomorphic liposarcoma and fibrosarcoma had large numbers of false positives among the ranked genes. However, the dedifferentiated/pleomorphic liposarcomas were characterized by a weak discriminatory 150-gene signature, which when used in a supervised cluster analysis grouped 5/12 dedifferentiated/pleomorphic liposarcomas tightly with 2 MFH/UPS, 2 leiomyosarcomas and 1 fibrosarcoma. Despite the high FDR, *CDK4*, *MDM1*, *MDM2*, *OS4 *and *SAS*, were among the top overexpressed and are along with several other highly expressed genes, e.g. *NUP107, SLC26A10*, *SLC35E3*, *TMBIM4*, *TSFM *and *YEATS4*, located in the 12q14 and 12q15 amplicons. EASE analysis also identified the above-mentioned gene group on chromosome 12q14-q15. Interestingly, also the MFH/UPS, the leiomyosarcomas and the fibrosarcoma that clustered tightly with the dedifferentiated/pleomorphic liposarcomas showed amplification of *CDK4 *and *MDM2 *when analyzed by array comparative genomic hybridization (data not shown) or Southern blot analysis [23]. In addition, genes involved in receptor activity, signaling and lipid metabolism (some of which have previously been reported in liposarcoma) such as *ACAA2*, *ARSA*, *DHRS3*, *PDE3A*, and *PPARA*, were upregulated [see Additional files 17 and 18].

Golub-score analysis identified a relatively poor discriminatory signal of 200 genes for the fibrosarcomas, which regardless of high FDR contained several of the upregulated genes previously associated with fibrosarcoma, e.g. *BMI1*, *H1F0*, *LEF1*, *RBM4*, *ITM2A*, *IGFBP2 *and *PTGS2 *[10]. Upregulation of developmental genes like *BMP7*, *SMO*, *VANGL2*, *SFRP1*, *PRRX1*, *MDK*, *OLFM*, *IGFBP3*, *IGFBP5 *and *TGFBR3 *in the fibrosarcoma samples suggests similarity to SS, GIST, myxoid/round-cell liposarcoma and MPNST explaining its classification within subcluster S in Figure 1 [see Additional files 19 and 20]. This shows that FDR, though important, cannot be taken at face value without the risk of losing biologically relevant information, especially in the case of STS where high FDR may result not only from heterogeneity and errors in diagnosis but also due to common pathogenic pathways resulting in similarities or overlap of expression profiles between different STS subtypes.

#### Myxoid/round-cell liposarcoma vs. Dedifferentiated/pleomorphic liposarcoma

In an independent analysis (including 16 tumor and 3 xenograft samples), the 5 myxoid/round-cell liposarcomas were compared to the 14 dedifferentiated/pleomorphic liposarcomas. A 800-gene signature (11% FDR) distinguished the two groups [see Additional file 2]. Developmental genes including members of the Wnt receptor signaling pathway, e.g. *DAAM1*,* FZD8*, *MYC*, *PRICKLE1*,* SFRP1 *and *WISP2*, and neurogenesis genes, e.g. *CPNE6*, *EFNA5*, *FEZ2*, *LHX2*, *MDK *and *NTNG1 *were upregulated in the myxoid/round-cell liposarcomas as compared to the dedifferentiated/pleomorphic liposarcomas, as were several ribosomal protein genes and genes involved in adhesion and amino acid metabolism. Genes highly expressed in the dedifferentiated/pleomorphic liposarcomas included cell-cycle genes like *CCNA2*,* CCNB2*, *CDC2*, *KIFC1*, *KIF23 *and *PTTG1*, motility genes like *AMFR*, *ANXA1*, *CKB*, *CNN2 *and *FN1 *and homeostasis-related genes. A smaller number of lipid metabolism genes were overexpressed in the myxoid/round-cell liposarcomas compared to the dedifferentiated/pleomorphic liposarcomas with genes such as *DGKD*,* EBPL*, *FABP5*, *FDFT1*, *LPL *and *PPARG *upregulated in the former group, while *ADM*, *ANXA1*, *ANXA4*, *CRYL1*, *GRN*, *PLA2G4A*, *PLA2G12A*, *PLD1 *and *PLTP *were overexpressed in the latter [see Additional files 2 and 21]. Similar results were obtained even with the exclusion of the 3 xenograft samples further supporting the notion that xenografts reflect gene expression patterns of patient tumor material and can with some caution be included in gene expression studies of STS to increase sample size for rare tumor types.

### Prognostic Signature

About 50% of the patients diagnosed with STS succumb to their disease owing partly to the high metastatic potential of these tumors but risk-assessment is rather difficult with very few reliable prognostic factors [24]. The different STS subtypes are associated with variable outcome with a favorable prognosis for patients with myxoid liposarcomas and a high risk of metastases for patients with MFH/UPS and SS [25]. Since the majority (76%) of the tumors in our series were high-grade, pleomorphic STS, we chose to evaluate the presence of a prognostic signature within subcluster C among the most heterogeneous samples. After excluding local recurrences, metastases, samples treated with preoperative chemotherapy, the single SS and 3 MPNST (that were outliers from cluster S), the 11 leiomyosarcomas with a distinct profile, and the dedifferentiated/pleomorphic liposarcomas with the *MDM2-CDK4 *double amplification, 89 primary STS remained for the analysis. Exclusion of the leiomyosarcomas and liposarcomas that formed subclusters within C was done in order to detect the more subtle prognostic signal among the most pleomorphic samples (Table 1).

**Table 1:** Summary of the clinicopathological data of the 177 and 89 STS samples

**Factor**	**Diagnostic signature n = 177**	**Prognostic signature n = 89**
**Sex **(male/female)	88/89	50/39
**Age, **median (range) years	66 (11 – 94)	69 (33 – 93)
**Histological subtypes**		
Malignant fibrous histiocytoma (MFH)	61^¤^	54
Leiomyosarcoma	40	27
Synovial sarcoma	32	0
Liposarcoma	16*	0
Malignant peripheral nerve sheath tumor	8	0
Myofibroblastic sarcoma	5	5
STS not otherwise specified	4	2
Extraskeletal osteosarcoma	3	1
Fibrosarcoma	3	0
Gastrointestinal stromal tumors	3	0
Epithelioid sarcoma	1	0
Malignant mesenchymoma	1	0
**Grade**		
II	6	1
III	32	12
IV	139	76
**Tumor size**		
Median (range) cm	8 (1–40)	9 (2–30)
< 5 cm	41	19
5 – 10 cm	74	38
>10 cm	62	32
**Tumor location**		
Extremity	144	78
Trunk wall	19	7
Retroperitoneum	7	3
Other	7^#^	1
**Tumor depth**		
Superficial	37	22
Deep	118	63
Unclassified	22	4
**Necrosis**		
Absent	48	23
Present	106	66
Unclassified	23	0
**Vascular invasion**		
Absent	121	72
Present	31	17
Unclassified	25	0
**Treatment**		
Surgery alone	107	58
Postoperative radiotherapy	51	22
Postoperative chemotherapy	3	2
Postoperative radio- and chemotherapy	10	7
Preoperative radio- or chemotherapy	6	0
**Surgery**		
Wide	90	57
Marginal	66	28
Intralesional	15	4
Unclassified	6	0

^¤^Includes 47 storiform, 13 myxoid, and 1 giant-cell MFH

* Includes 4 myxoid/round cell liposarcomas, 6 dedifferentiated liposarcomas, and 6 pleomorphic liposarcomas

^# ^Includes localizations in the abdomen, the mediastinum, and the head and neck

#### Supervised analysis

**Figure 2:**
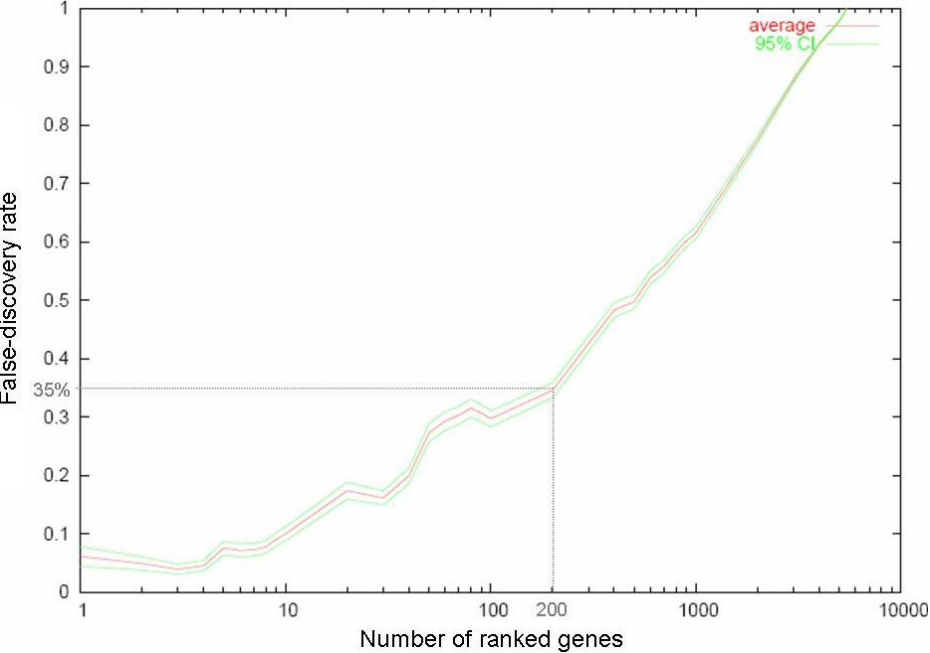
Plot showing FDR within the Golub-score ranked prognostic genes distinguishing the primary tumors that developed metastasis from those that remained metastasis-free. The number of ranked genes is plotted along the x-axis and FDR along the y-axis.

**Figure 3:**
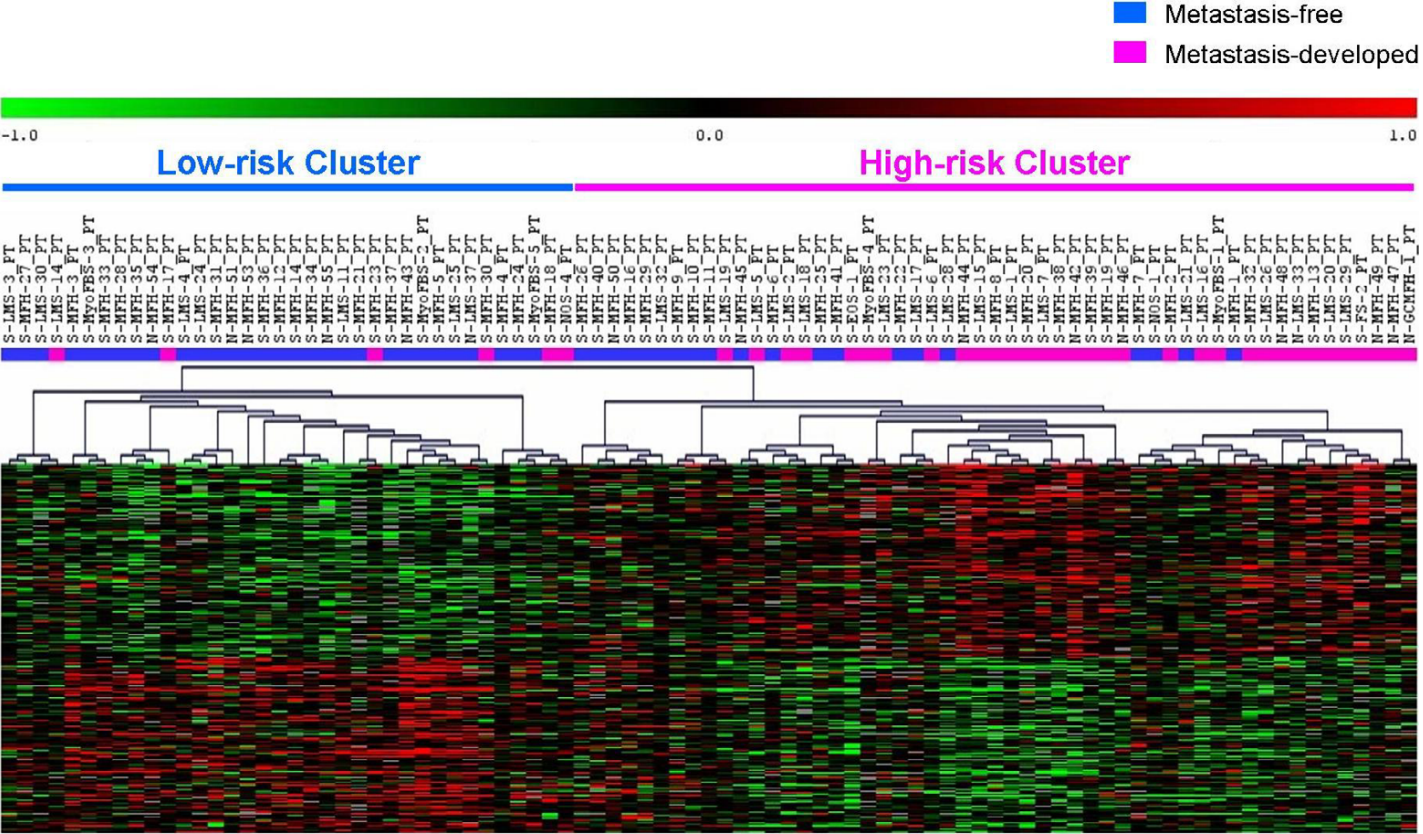
Supervised clustering of the 89 primary pleomorphic STS samples based on the 244-gene prognostic signature.

Golub-score analysis with 1000 random permutations identified a 200-gene prognostic signature (35% FDR) distinguishing tumors that metastasized (n = 39) from those that remained metastasis-free (n = 50) (Figure 2). In order to obtain a more robust list of discriminators, a consensus gene list of 244 genes with median rank less than 700 was generated, as described in the methods section, with the majority of the genes from the Golub-score ranked list being present in the consensus list. Hierarchical clustering based on the consensus list split the 89 samples into two clusters with metastases developing in 6/36 (16%) in the low-risk cluster compared to 33/53 (62%) in the high-risk cluster (Figure 3). The genes overexpressed in the metastasizing tumors included *HYOU1*, *HIF1A*, *HIG2*, *DDIT4*, *TFRC*, *ERO1L*, *PLOD2*, and *ADM *suggesting an expression program triggered by hypoxia. Hypoxia causes stabilization of the HIF-1 transcription factor that mediates the induction of several genes including those promoting anaerobic glycolysis [26] and the most significant functional group identified in the EASE analysis included glycolytic enzymes and glucose transporters like *ENO1*, *ENO2*, *PYGL*, *FUT1*, *HK2*, *GLUT1*, *GYS1*, *PDK1*, *CA2*, *CA12*, *PGK1 *and *LDHB*, many of which are known markers for hypoxia. The overexpression of hypoxia-induced genes in metastasizing primary tumors provides a basis for further studies of hypoxia in STS to clarify its role in metastasis and to verify potential prognostic and therapeutic utility. In addition, several genes involved in cell proliferation, adhesion and motility e.g. *SYMPK*, *ACTN1*, *BYSL*, *VCL*, *NRCAM*, *YARS *and *TLN1 *were among the discriminators [see Additional file 22].

#### Support vector machine (SVM) leave-one-out cross-validation and statistical analyses

**Figure 4:**
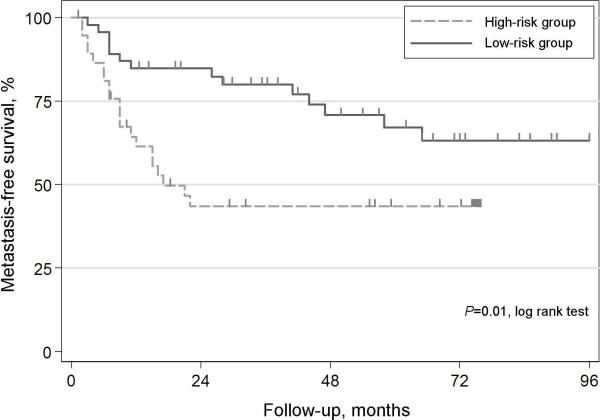
Kaplan-Meier estimates of metastasis-free-survival for patients included in the prognostic subset (5 cases with metastasis at diagnosis were excluded) classified as high-risk or low-risk by the SVM cross-validated classifier.

Development of metastasis correlated with tumor size (*P *= 0.006, Mann-Whitney's U and Kruskal-Wallis tests) and necrosis (*P *= 0.013, Pearson χ^2 ^test), but not with vascular invasion (*P *= 0.166, Pearson χ^2 ^test). The SVM leave-one-out cross-validation correctly classified 64% of the samples (area under receiver operating characteristic (ROC) curve = 0.64, *P *= 0.007) into two groups with metastasis developing in 58% (25/43) of the patients in the high-risk group, compared to 30% (14/46) in the low-risk group (*P *= 0.008, Pearson χ^2 ^test) and significantly predicted metastasis-free survival (*P *= 0.01, logrank test, Figure 4). The corresponding hazard ratio (HR) from a univariate Cox-regression analysis was 2.4 (*P *= 0.01) and in a multivariate analysis including the established prognostic factors size, necrosis and vascular invasion, the profile predicted outcome with a HR of 2.2 (*P *= 0.04) (Table 2).

**Table 2:** Univariate and multivariate analysis in the prognostic subset of 89 primary tumors

			**Metastasis-free survival**			
			
**Factors**	**Total Number**	**Metastasis-free/Metastasis developed**	**Univariate analysis**		**Multivariate analysis**	
				
			**HR (95% CI)**	***P *-value**	**HR (95% CI)**	***P *-value**
**Number of samples**	89	50/39				
**Tumor size**						
Median (range) cm	9 (2 – 30)	7.5 (2 – 28)/10 (3 – 30)	1.1 (1.02 – 1.12)	0.012	1.2 (1.01–1.14)	0.021
**Necrosis**						
Absent	23	18/5	1.0			
Present	66	32/34	2.9 (1.12 – 7.50)	0.028	1.6 (0.55–4.52)	0.402
**Vascular invasion**^*a*^						
Absent	72	43/29	1.0			
Present	17	7/10	2.4 (1.11 – 5.17)	0.026	2.2 (0.98–5.00)	0.055
**Cross-validated classifier**^*a*^						
low-risk group	46	32/14	1.0			
high-risk group	43	18/25	2.4 (1.20 – 4.73)	0.013	2.2 (1.04–4.62)	0.04

^*a*^Proportional hazards assumptions assume constant mortailty ratios and are therefore not met. Thus, the estimated HRs should be interpreted as averages over time. The effects are considerably larger initially and level off with time.

## Discussion

From a clinical diagnostic perspective, most STS with specific fusion proteins can be correctly classified based on the combined data from histopathology, immunostainings, and cytogenetic and/or molecular genetic analyses. This stands in contrast to the genetically complex STS subtypes, the diagnosis of which is hampered by suboptimal reproducibility due to extensive histopathological heterogeneity, pleomorphism and lack of type-specific genetic defects. Distinct and homogenous expression profiles have been reported in SS, dermatofibrosarcoma protuberans, clear-cell sarcoma, myxoid/round-cell liposarcoma and GIST [4-13]. Furthermore, the discriminatory profiles that often contain genes located downstream of the type-specific pathogenic gene fusions or mutations have provided novel targets for molecular therapy [15-18]. Gene expression profiles have provided clues to the histogenesis of some STS subtypes, suggested similarities between others and allowed distinction between pathologically inseparable lesions, e.g. neural crest origin of SS, close relation between clear-cell sarcoma and melanoma, and distinct expression profiles for well-differentiated and dedifferentiated liposarcomas [9,11,27]. Current data from the more pleomorphic STS, however, indicate that their expression profiles are indeed heterogeneous and in these tumor types the primary goal may rather be to identify novel, yet unidentified subgroups, and to clarify deregulated genes and pathways.

Unsupervised cluster analysis of the 177 STS identified two major subclusters; S containing STS with specific fusion genes or mutations, e.g. SS, myxoid/round-cell liposarcoma and GIST, and subcluster C with genetically complex, often pleomorphic STS subtypes, e.g. MFH/UPS, leiomyosarcoma and dedifferentiated/pleomorphic liposarcoma (Figure 1). This is in line with results from previous gene expression and proteomic profiling studies [10,19,28].

Discrimination scoring identified genes and pathways differentially regulated in the STS subtypes, with considerable amount of overlap with previously published gene lists despite the differences in tumor material and array platforms [4,5,7,9,10,12,19,20]. Expression profiles in the distinct subtypes were strong with low FDR whereas the pleomorphic ones had relatively heterogeneous profiles with higher FDR. Identification of diagnostic markers that clearly distinguish different subtypes requires gene lists with very low FDR, but inherent heterogeneity, lack of clear-cut boundaries between subtypes, misdiagnoses and common pathogenic genes and pathways make it rather difficult to generate such strong diagnosis-specific expression profiles in the pleomorphic STS. Some studies chose to exclude samples that did not cluster according to histopathological diagnosis thereby reducing FDR by increasing homogeneity within the subgroups [7,10]. In the current study, outliers were included but lenient cut-offs were employed allowing some amount of false positives in the gene lists, which were then analyzed using EASE to identify functionally correlated genes. The risk of signifying false positives was reduced by focusing on upregulated pathways and functional groups rather than on individual genes. This approach helped identify biologically relevant genes in dedifferentiated/pleomorphic liposarcoma and fibrosarcoma despite high FDR and upregulation of similar functional groups in SS, GIST, myxoid/round-cell liposarcoma, MPNST and fibrosarcoma.

Although alternative *SSX *fusions and various lines of differentiation can be demonstrated in SS [29,30], they display homogenous and distinct expression profiles that enable clear distinction from other STS subtypes [4,9,10,12,19,20] and our finding of 4000 differentially expressed genes is in line with these results. In the unsupervised clustering, all but one SS were part of subcluster S, within which 5 formed a tight subcluster, together with 3 MPNST samples on a separate branch, away from the main SS subcluster (Figure 1). Supervised clustering based on the 4000 genes also showed close clustering of SS to MPNST [see Additional file 5]. Previous studies have shown that SS and MPNST share similar patterns of gene expression with upregulation of neuroectodermal differentiation genes thereby suggesting a neural crest origin [4,7,9] and several neural differentiation genes were also upregulated in the present study. The SS18-SSX fusion product controls gene expression by association with chromatin remodeling complexes through interactions with SWI/SNF complexes and histones [31-33]. Interestingly, chromatin-remodeling genes including several histones and SMARC genes constituted one of the largest functional groups upregulated in the SS. Moreover, the genes identified herein verified the significance of developmental pathways including the FGF, EGF, TGF-β, Wnt, Notch, retinoic acid and Hh receptor signaling pathways, the targeted inhibition of which may provide novel therapeutic options.

MPNST may morphologically mimic SS [34] and in the unsupervised analysis, 5/8 MPNST clustered within subcluster S, and 3 with a subset of SS. Several developmental, neural differentiation and ribosomal protein genes were found to be overexpressed in both MPNST and SS. Fibrosarcomas, like the MPNST, have been shown to cluster with the SS [7,10]. The fibrosarcomas in this study were part of subcluster S and a weak expression signature including many developmental genes distinguished these tumors from the remaining STS.

GIST constitute a distinct STS subtype, ~80% of which carry *KIT *gene activating mutations [35]. These tumors demonstrate distinct expression profiles [5], with upregulation of genes within the *KIT *signaling pathway. In addition, several genes involved in neurogenesis and neural differentiation were overexpressed, as were muscle-specific genes like smoothelin and myosin, which may reflect the suggested origin of GIST from the interstitial pacemaker cells of Cajal [36].

Liposarcomas account for about 20% of STS and within this subtype, the myxoid/round-cell liposarcomas characterized by the *TLS-CHOP *or *EWS-CHOP *fusions [37] formed a distinct cluster closely related to a subset of SS and MPNST within subcluster S with upregulation of several developmental and ribosomal protein genes (Figure 1). One of the top most upregulated genes was the lipid metabolism gene *PPARG *that regulates adipocyte differentiation and constitutes a potential therapeutic target [38,39]. The dedifferentiated/pleomorphic liposarcomas contain complex genetic alterations and were scattered among the pleomorphic samples in the unsupervised clustering. Most dedifferentiated liposarcomas are characterized by amplifications of *MDM2 *and *CDK4 *as part of the 12q amplicons involved in the formation of ring-chromosomes [40,41]. A relatively weak discriminatory signature mainly characterized by overexpression of the 12q amplicon genes distinguished a small subset of the dedifferentiated/pleomorphic liposarcomas [see Additional file 17]. *PPARA *and other lipid metabolism genes were also overexpressed. Comparison between myxoid/round-cell and pleomorphic/dedifferentiated liposarcomas revealed overexpression of ribosomal proteins and developmental genes related to Wnt signaling and neurogenesis in the former group, whereas genes related to cell-cycle, homeostasis, and a greater number of lipid metabolism genes were upregulated in the latter. Skubitz *et al*. demonstrated separation of myxoid liposarcoma from non-myxoid liposarcoma using a set of ribosomal genes [42].

Our series included a large proportion of pleomorphic, undifferentiated STS with the hope of gaining novel insights into the origin of these genetically complex tumors and establishing a more refined classification. About one-third of the leiomyosarcomas clustered tightly within subcluster C and a 200-gene signature with overexpression of genes related to muscle structure and function was identified in these tumors (Figure 1). This is in line with previous results from gene expression and proteomic profiling demonstrating that while the more pleomorphic leiomyosarcomas cluster with the MFH, another more distinct subset cluster separately [19,28]. MFH was introduced as a separate diagnostic entity in the 1960s, but constitutes a heterogeneous group of poorly differentiated tumors with poor diagnostic reproducibility [1]. In our series, one-third of the tumors represented MFH/UPS, all but 5 of which fell within subcluster C (Figure 1). A 500-gene signature grouped together about half of the MFH/UPS [see Additional file 15] including several genes from the extracellular matrix and inflammatory response, reflecting the fibrous and histiocytic features of these tumors, as well as others involved in the regulation of cell-cycle, proliferation, adhesion, motility and protein degradation.

Novel prognostic and therapeutic markers would be of great clinical value in STS since risk stratification is currently difficult and adjuvant treatments are toxic and hampered by low efficacy. We identified a 244-gene prognostic signature in 89 primary, high-grade STS mainly representing MFH/UPS and leiomyosarcoma. This signature was characterized by upregulation of several hypoxia-related genes (e.g. *HIF1A *and its targets) and genes involved in cell proliferation, adhesion and motility in metastasizing STS. Our data are the first to suggest a prognostic profile modulated at least in part by hypoxia in a large series of highly malignant STS of mixed types. The cross-validated classifier predicted metastasis with an accuracy of 64% and provided prognostic information independent of currently used prognostic factors from the SIN-system, namely tumor size, vascular invasion, and necrosis [43]. Gene expression profiles that correlate with poor outcome have previously been recognized in Ewing sarcoma and leiomyosarcoma [21,22,44], Ren *et al*. identified a 92-gene signature in 11 leiomyosarcomas that separated high-grade metastatic tumors from low-grade well-differentiated ones, whereas Lee *et al*. took a different approach and predicted metastasis in a set of 30 primary leiomyosarcomas and local recurrences using the expression profile of 335 genes that initially distinguished primary leiomyosarcomas from metastases [21,22]. However, none of the reported genes were among our 244 discriminators, which may not only be because they were established in a set of leiomyosarcomas using different approaches whereas our signature was established in a larger mixed series of pleomorphic primary STS, but also due to the difficulties in identifying prognostic signals which are considerably weaker than the diagnostic ones.

An adverse prognostic impact of hypoxia has been demonstrated in several malignancies and tumor oxygenation studies in STS have suggested an association of hypoxia with tumor grade, presence of mitoses and metastatic development [45-48]. A recent study demonstrated that HIF1A expression was an independent prognostic factor in STS [49]. Hypoxic tumors display high rates of glucose uptake and glycolysis regulated by HIF1 that induces expression of glucose transporters like GLUT1 and glycolytic enzymes like ENO1, HK2, LDHB and PGK1[50], which were overexpressed in the metastasizing tumors. CA9 expression, an intrinsic cellular marker for hypoxia, has been suggested to correlate with poor survival in high-grade STS [51], and GLUT1 expression and enhanced glucose metabolism in STS have been linked to proliferative activity and tumor grade [52-55]. Detwiller *et al*. demonstrated that the expression pattern of a selection of 107 hypoxia-related genes allowed distinction of mixed STS samples from normal tissue, and several of the overexpressed genes therein were among our discriminators [56].

## Conclusion

In summary, diagnostic gene expression profiles were identified for different subtypes with distinct profiles in STS with specific fusion genes or mutations, whereas the diagnostically difficult pleomorphic STS were challenging also with regards to expression profiling. The SS, GIST, myxoid/round-cell liposarcomas, MPNST and fibrosarcomas shared similarities in expression profiles with overrepresentation of developmental genes involved in differentiation and morphogenesis. On the other hand, genes involved in cell-cycle, proliferation, adhesion, motility, protein degradation, homeostasis and immune-response seemed to play an important role in the pleomorphic subtypes. The novel genes and pathways identified provide important information about tumor origin and constitute potential therapeutic targets. Our identification of a prognostic profile in the latter group is highly promising, and provides information independent of the currently used prognosticators. Moreover, it is intriguing that upregulation of hypoxia-related genes predicts metastatic potential in high-grade, pleomorphic and genetically complex STS and calls for further evaluation of HIF1A and its target genes in STS.

## Methods

### Patients and tumor material

Tumor samples were obtained from 177 patients operated between 1972 and 2003 at the Lund University Hospital, Lund (n = 122), the Norwegian Radium Hospital, Oslo (n = 47) and the Karolinska Hospital, Stockholm (n = 8). Ethical permission for the study was obtained from the Lund University research ethics committee and the Regional Ethics Committee of Southern Norway. The tumor samples consisted of 154 primary tumors, 16 local recurrences and 7 metastases, and the latter samples were included after assuring that neither local recurrences nor metastases formed separate clusters (data not shown). The 177 samples represented 13 subtypes, among which MFH/UPS, leiomyosarcoma and SS together constituted 75% (Table 1). All tumors were reviewed by the Scandinavian Sarcoma Group (SSG) review board of pathologists and many tumors had in addition been reviewed by one of two reference pathologists (BB & MÅ), who also belonged to the SSG board of pathologists. The diagnoses were based on the combined information from histopathology, immunohistochemical staining, and cytogenetic and/or molecular genetic analyses. The *SS18-SSX *gene fusion was confirmed in 24/32 synovial sarcomas (13 with SS18-SSX1, 10 with SS18-SSX2 and 1 with SS18-SSX4), and the *TLS-CHOP *fusion in 2/4 myxoid/round-cell liposarcomas. The 8 MPNST were all derived from patients free of neurofibromatosis. Malignancy grading was based on a IV-tiered scale, and in line with our aim to improve diagnosis of high-grade tumors, 97% of the tumors were classified as high-grade (grades III and IV). Only a small minority of the patients received preoperative treatment with radiotherapy (n = 1) or chemotherapy (n = 5). The study is aimed at tumors located in the extremities and the trunk wall, but 14/177 (8%) abdominal/retroperitoneal tumors were included since they contributed with data on rare tumor types and did not cluster separately (data not shown). Besides the 177 patient samples, 17 xenografts (including 6 MFH/UPS, 4 MPNST, 3 SS, 2 pleomorphic liposarcomas, 1 myxoid/round-cell liposarcoma and 1 GIST) were included, but were not used for the generation of discriminatory gene lists. Analysis of a prognostic expression profile was performed in 89 primary pleomorphic tumors, mostly including MFH/UPS and leiomyosarcomas. The prognostic system used for clinical decisions included evaluation of necrosis and vascular invasion. Necrosis was classified at histopathological examination either if identified macroscopically or when identified microscopically at careful examination. Necrosis was identified in 66/89 (74%) tumors in the prognostic subset, thus at a high rate. Vascular invasion also carefully examined for prognostic purposes was identified in 17/89 (19%) of the tumors (Table 1). All 89 patients had undergone primary surgery without preoperative radio- or chemotherapy and only 9 patients were treated with postoperative chemotherapy. Metastasis developed in 39 (44%) patients after median 9 (range 0–65) months and the mean follow-up for the survivors was 7 (range 1–16) years.

### RNA extraction and cDNA microarray analysis

RNA extractions from 47 tumors and the 17 xenografts were carried out at the Norwegian Radium Hospital, whereas the remaining 130 samples were extracted at the Lund University Hospital, and all 194 samples were labeled and hybridized in Lund. The cDNA microarray slides used were produced at the Swegene DNA Microarray Resource Center, Department of Oncology, Lund University and contained 27649 spots with sequence-verified IMAGE clones from the Research Genetics IMAGE clone library. The clone information was linked to gene names using build 180 of the Unigene database [57] and ~16000 unique Unigene clusters were represented on the array. The RNA extraction, cDNA synthesis, labeling, hybridization and subsequent image and data processing were carried out as previously described [58]. Background correction, filtering, transformations and analyses were performed using a local installation of the web-based BioArray Software Environment (BASE) [59,60]. A preliminary filtering step eliminated all spots of poor quality like those flagged 'not found' or 'bad' in GenePix™ Pro 4.1.1.4 version (Axon instruments Inc., Foster City, CA), spots with diameter lesser than or equal to 60 μm, spots with more than 10% pixel saturation and signal-to-noise ratio less than 1.5 in either channel. The background corrected intensity values were then normalized using the pin-based LOWESS method to compensate for dye bias and local background effects [61]. Here, intensity dependent adjustments (LOWESS fits) were performed within groups of 16 blocks to correct for spatial bias. Multiple print batches of slides were used, with 41 samples hybridized in replicates on different batches. All repeats clustered next to the first sample run irrespective of the differences in print batch (data not shown), hence replicate assays were merged in a weighted fashion, as previously described [58]. Within each slide, expression values for spots associated to the same gene symbol were merged in a similar weighted fashion. A student's t-test identified ~2500 genes (*P *= 0.05) that differed in expression between samples extracted in Norway and Sweden and ~1800 genes (*P *= 0.05) that differed between tumors and xenografts suggesting the presence of technical and biological bias within the data set. In order to eliminate the technical bias introduced by the RNA extractions carried out at different laboratories, centering was applied independently to the 64 (tumors and xenografts) Norwegian and 130 Swedish samples. The xenograft samples, wherever used in the analysis, were centered separately a second time to compensate for the inherent biological differences that exist between tumors and xenografts. Following independent centering of the mentioned sample groups, the student's t-test failed to identify significant genes that distinguished the groups confirming that very little, if any, of the bias remained. Moreover, in later steps no apparent clustering was observed based on RNA extraction or depending on whether the sample was from a tumor or xenograft. The data were then transformed using an error model, as previously described, to reduce the importance of poor-quality spots in later analysis steps [58]. Filters for variation and presence of expression across hybridizations were set to reject all spots with a standard deviation of modified expression value smaller than 0.2 and a presence in less than 70% of the samples. Unsupervised agglomerative hierarchical clustering was performed with the help of the TMeV application from the TM_4 _microarray software suite [62], using the average-linkage method and the Pearson correlation distance metric [63].

Golub-score analyses, named after the widely referenced paper by Golub *et al*. [64], and random permutations were performed as previously described [58] on 177 and 142 (excluding SS and GIST) tumor samples in order to generate discriminatory gene lists for the different subtypes. The SS and GIST, which revealed distinct expression patterns, were excluded in order to identify the more subtle differences in genetic profiles of the remaining STS subtypes. Approximately 6000 genes were ranked on their Golub-scores – a high score for a gene implicating minor variation in expression within the subtype but large variation between the subtypes, in turn implicating high discriminating power. 1000 random permutations were performed to assess the discriminating power of the scores and to establish FDR. Furthermore, the 19 liposarcoma samples (16 tumors and 3 xenografts) were analyzed independently to identify a genetic signature distinguishing the myxoid/round-cell liposarcomas from the dedifferentiated/pleomorphic liposarcomas.

Golub-score analysis was also used to identify a metastatic signature within a subset of 89 primary, mainly high-grade, pleomorphic tumors. To obtain a more robust list of prognostic discriminators, a consensus gene list was created. The 89 samples, 39 of which metastasized, were randomly split into two halves preserving the ratio of metastasizing samples and each half was used to create two Golub-score ranked gene lists. The above step was iterated 100 times to obtain 200 gene lists in total, from which a consensus gene list was established by ordering genes according to median rank. The top 244 genes (median rank < 700) were used to cluster the samples in TMeV. Thereafter, leave-one-out cross-validation using the SVM option in TMeV was performed on the 89 samples based on all ~5500 genes that passed the above-mentioned filter criteria in order to obtain an unbiased classification that was later used in the univariate and multivariate analyses.

The discriminatory gene lists were further analyzed using the EASE software [65] to functionally classify the genes and facilitate biological interpretations [66]. The top ranked genes were classified into groups within the categorical systems of the Gene Ontology (GO) Consortium (GO Biological Process and GO Molecular Function), the KEGG pathway, biochemical process, cellular role and chromosomal regions. The EASE analyses in SS and myxoid/round-cell liposarcomas employed the top 4000 and 1000 genes (with 11% FDR) respectively. Gene lists with less stringent cut-offs were used for the GIST (top 1500 with 25% FDR), MFH/UPS (top 500 with 16% FDR), leiomyosarcomas (top 500 with 26% FDR) and MPNST (top 500 with 27% FDR), which allowed more genes into the gene lists making it possible to identify functional correlations between the discriminatory genes and similarities in expression profiles between the different subtypes. Focusing on upregulated pathways and functional groups rather than on individual genes reduces the risk of signifying biologically irrelevant genes, especially in gene lists with high FDR. All functional groups mentioned had an EASE score < 0.05.

### Statistical analyses

The χ^2 ^test for association, the Mann-Whitney's U test, and the Kruskal-Wallis test were used to assess associations of tumor size (as a continuous variable), necrosis (present *vs*. absent), vascular invasion (present *vs*. absent), and the SVM cross-validated classification with the development of metastasis. Metastasis-free survival curves were constructed by the Kaplan-Meier method and compared by the log-rank test. Univariate and multivariate Cox-regression analyses were performed to estimate HRs and to assess the independence of the cross-validated classification from the above-mentioned prognostic factors. Proportional hazards assumptions were checked using Schoenfeld's test [67]. Areas under ROC curves were compared using an algorithm suggested by DeLong *et al*. [68]. A two-tailed *P*-value of less than 0.05 was considered significant for all tests. Stata 9.2 was used for the statistical analyses (Stata Corporation, 2003, College Station, TX).

## Abbreviations

Soft tissue sarcoma: STS; Malignant fibrous histiocytoma: MFH; Undifferentiated pleomorphic sarcoma: UPS; Synovial sarcoma: SS; Malignant peripheral nerve sheath tumor: MPNST; Gastrointestinal stromal tumors: GIST; Not otherwise specified: NOS; False-discovery rates: FDR; Hedgehog: Hh; Retinoic acid receptor: RAR; EASE: Expression Analysis Systematic Explorer; SWI/SNF related matrix associated actin dependent regulator of chromatin: SMARC; Support vector machine: SVM; Receiver operating characteristic: ROC; Hazard Ratio: HR; Scandinavian Sarcoma Group: SSG; BioArray Software Environment: BASE; Gene Ontology: GO

## Authors' contributions

PF drafted the manuscript and carried out RNA extractions, hybridizations and data analyses. HMN performed extractions and hybridizations of the Norwegian samples and participated in the data analyses. CM assisted with clinical annotations of the Norwegian samples. PE designed the BASE plug-ins and assisted with the computational analyses. JF, JMB and AI performed RNA extractions and hybridizations. BB and MÅ were the pathologists who reviewed the tumor samples. POB performed the statistical analyses. AR participated in the design of the study and clinically annotated the Swedish samples. OM and MN conceived of the study, and participated in its design and coordination. All authors read and approved the final manuscript.

## Supplementary Material

Additional file 1 Unsupervised cluster analysis of the 194 STS samples including 17 xenografts. Figure **A **shows the unsupervised cluster analysis of the 194 STS samples where 2/3 synovial sarcoma xenografts and the single GIST and myxoid/round-cell liposarcoma xenografts clustered with their respective tumor histotypes, whereas all 6 MFH xenografts were part of the pleomorphic STS subcluster (the xenografts are indicated by red arrows). Figure **B **shows the same unsupervised cluster as above, this time showing the 3 xenografts derived from tumors included in the study (red arrows) that clustered next to their respective patient samples (blue arrows). Click here for file

Additional file 2 Independent analysis of the 19 liposarcoma samples including 16 patient samples and 3 xenografts. Figure **A **shows unsupervised cluster analysis of the 19 liposarcoma samples. The plot in figure **B **shows FDR within the Golub-score ranked genes distinguishing the myxoid/round-cell liposarcomas from the dedifferentiated/pleomorphic liposarcomas. The number of ranked genes is plotted along the x-axis and FDR along the y-axis. Figure **C **shows supervised clustering of the 19 liposarcoma samples based on the top 1000 genes discriminating the myxoid/round-cell liposarcomas from the dedifferentiated/pleomorphic liposarcomas. Click here for file

Additional file 3 FDR plots for the diagnostic signatures. FDR plots for the diagnostic signatures with the number of ranked genes plotted along the x-axis and FDR along the y-axis. Random permutation tests with 1000 permutations were performed to assess the discriminating power or robustness of the Golub-score ranked genes. For each rank, the average number of genes in a permutation list above that rank was divided by the number of genes in the true list to get the FDR. Click here for file

Additional file 4 Numbers and percentages of false positives among the top ranked genes. A table showing the numbers and percentages of false positives among the Golub-score ranked genes for the different discriminatory gene lists. Click here for file

Additional file 5 Supervised clustering based on the SS signature. Supervised clustering of 177 STS samples based on the top 4000 synovial sarcoma discriminating genes. Click here for file

Additional file 6 Top 4000 genes discriminating the synovial sarcomas from the remaining STS subtypes. The SS discriminators. Click here for file

Additional file 7 Supervised clustering based on the GIST signature. Supervised clustering of 177 STS samples based on the top 1500 GIST discriminating genes. Click here for file

Additional file 8 Top 1500 genes discriminating the GIST from the remaining STS subtypes. The GIST discriminators. Click here for file

Additional file 9 Supervised clustering based on the myxoid/round-cell liposarcoma signature. Supervised clustering of 142 STS samples based on the top 1000 myxoid/round-cell liposarcoma discriminating genes. Click here for file

Additional file 10 Top 1000 genes discriminating the myxoid/round-cell liposarcomas from the remaining STS subtypes. The myxoid/round-cell liposarcoma discriminators. Click here for file

Additional file 11 Supervised clustering based on the MPNST signature. Supervised clustering of 142 STS samples based on the top 500 MPNST discriminating genes. Click here for file

Additional file 12 Top 500 genes discriminating the MPNST from the remaining STS subtypes. The MPNST discriminators. Click here for file

Additional file 13 Supervised clustering based on the leiomyosarcoma signature. Supervised clustering of 142 STS samples based on the top 500 leiomyosarcoma discriminating genes. Click here for file

Additional file 14 Top 500 genes discriminating the leiomyosarcomas from the remaining STS subtypes. The leiomyosarcoma discriminators. Click here for file

Additional file 15 Supervised clustering based on the MFH/UPS signature. Supervised clustering of 142 STS samples based on the top 500 MFH/UPS discriminating genes. Click here for file

Additional file 16 Top 500 genes discriminating the MFH/UPS from the remaining STS subtypes. The MFH/UPS discriminators. Click here for file

Additional file 17 Supervised clustering based on the dedifferentiated/pleomorphic liposarcoma signature. Supervised clustering of 142 STS samples based on the top 150 dedifferentiated/pleomorphic liposarcoma discriminating genes showing 5/12 dedifferentiated/pleomorphic liposarcomas clustered tightly with 2 MFH/UPS, 2 leiomyosarcomas and 1 fibrosarcoma, all of which possess the *CDK4-MDM2 *amplification. Click here for file

Additional file 18 Top 150 genes discriminating the dedifferentiated/pleomorphic liposarcomas from the remaining STS subtypes. The dedifferentiated/pleomorphic liposarcoma discriminators. Click here for file

Additional file 19 Supervised clustering based on the fibrosarcoma signature. Supervised clustering of 142 STS samples based on the top 100 (44% FDR) and top 200 (50% FDR) fibrosarcoma discriminating genes. Click here for file

Additional file 20 Top 200 genes discriminating the fibrosarcomas from the remaining STS subtypes. The fibrosarcoma discriminators. Click here for file

Additional file 21 Top 1000 genes discriminating the myxoid/round-cell liposarcomas from the dedifferentiated/pleomorphic liposarcomas. The genes distinguishing the myxoid/round-cell liposarcomas from the dedifferentiated/pleomorphic liposarcomas in an independent analysis of 19 liposarcoma samples including 3 xenografts. Click here for file

Additional file 22 Top 244 genes sorted on median rank discriminating the tumors that developed metastasis from the ones that were metastasis-free. The metastasis signature established in 89 high-grade pleomorphic primary tumors. Click here for file
