# Supplementary figures and images for: Diagnostic and prognostic gene expression signatures in 177 soft tissue sarcomas: hypoxia-induced transcription profile signifies metastatic potential
Source: BMC Genomics. 2007 Mar 14;8:73. doi: 10.1186/1471-2164-8-73 (PMC1839099; doi:10.1186/1471-2164-8-73)

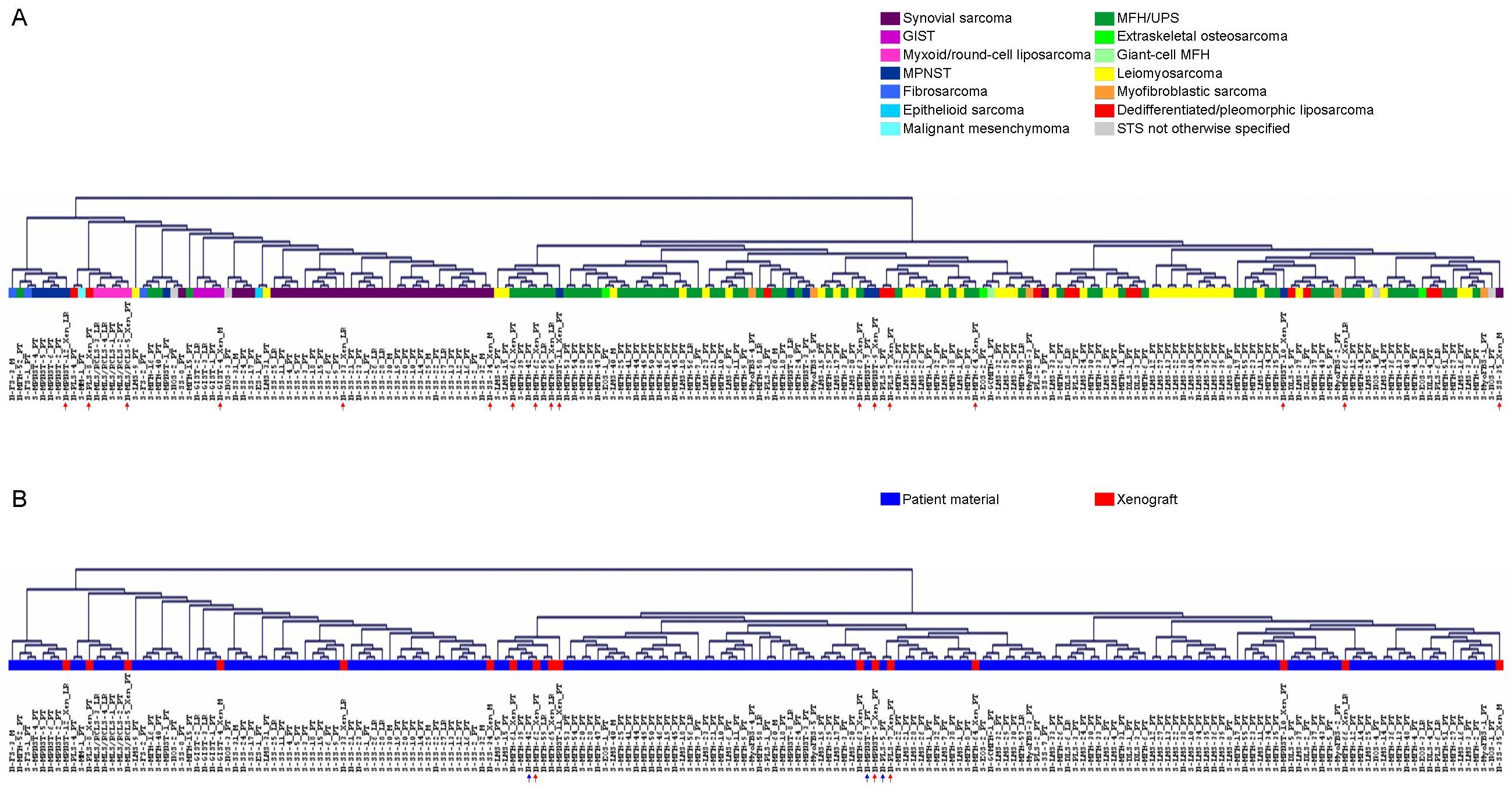

Supplement: Additional file 1 — Unsupervised cluster analysis of the 194 STS samples including 17 xenografts. Figure A shows the unsupervised cluster analysis of the 194 STS samples where 2/3 synovial sarcoma xenografts and the single GIST and myxoid/round-cell liposarcoma xenografts clustered with their respective tumor histotypes, whereas all 6 MFH xenografts were part of the pleomorphic STS subcluster (the xenografts are indicated by red arrows). Figure B shows the same unsupervised cluster as above, this time showing the 3 xenografts derived from tumors included in the study (red arrows) that clustered next to their respective patient samples (blue arrows). [file 1471-2164-8-73-S1.jpeg]

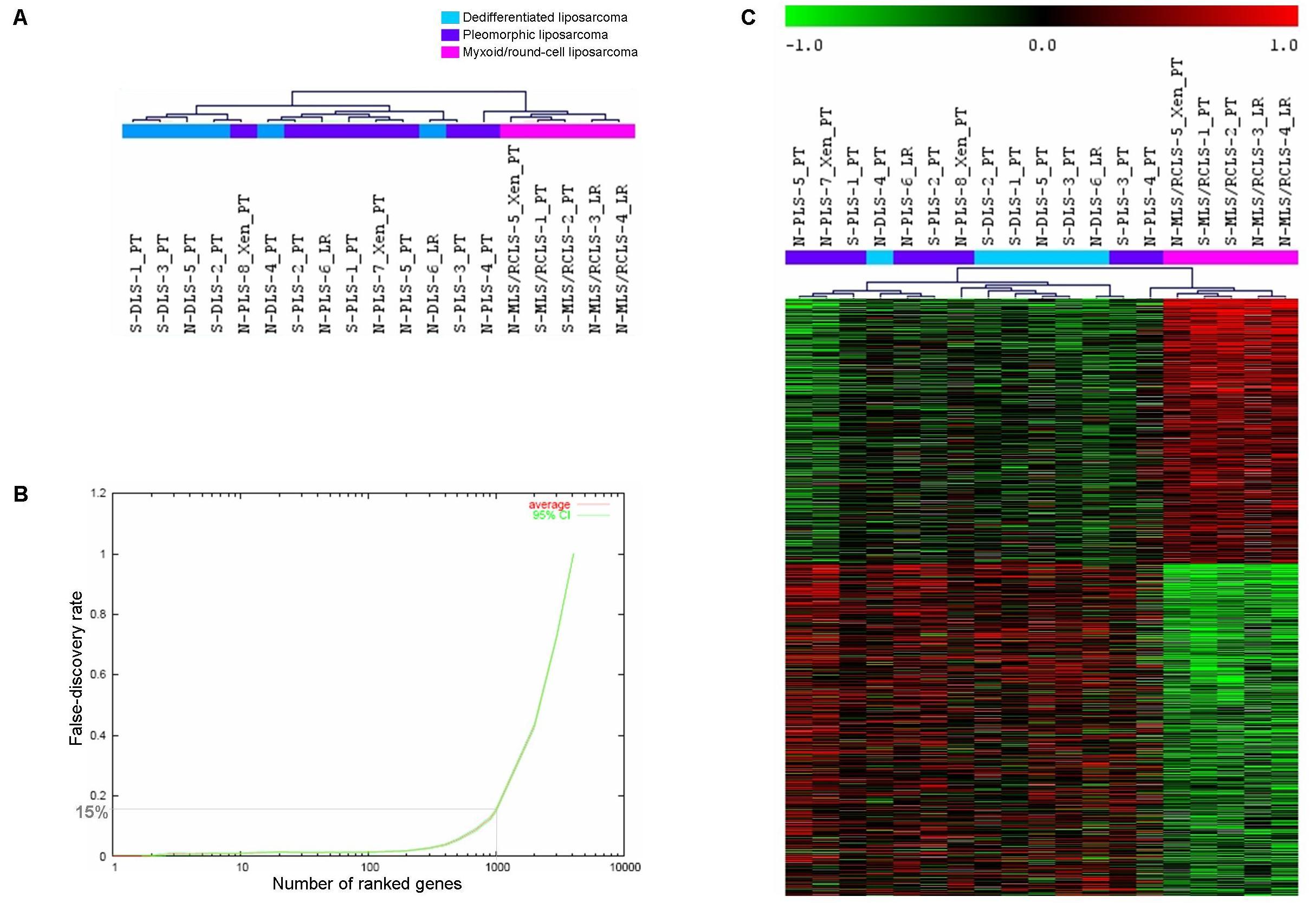

Supplement: Additional file 2 — Independent analysis of the 19 liposarcoma samples including 16 patient samples and 3 xenografts. Figure A shows unsupervised cluster analysis of the 19 liposarcoma samples. The plot in figure B shows FDR within the Golub-score ranked genes distinguishing the myxoid/round-cell liposarcomas from the dedifferentiated/pleomorphic liposarcomas. The number of ranked genes is plotted along the x-axis and FDR along the y-axis. Figure C shows supervised clustering of the 19 liposarcoma samples based on the top 1000 genes discriminating the myxoid/round-cell liposarcomas from the dedifferentiated/pleomorphic liposarcomas. [file 1471-2164-8-73-S2.jpeg]

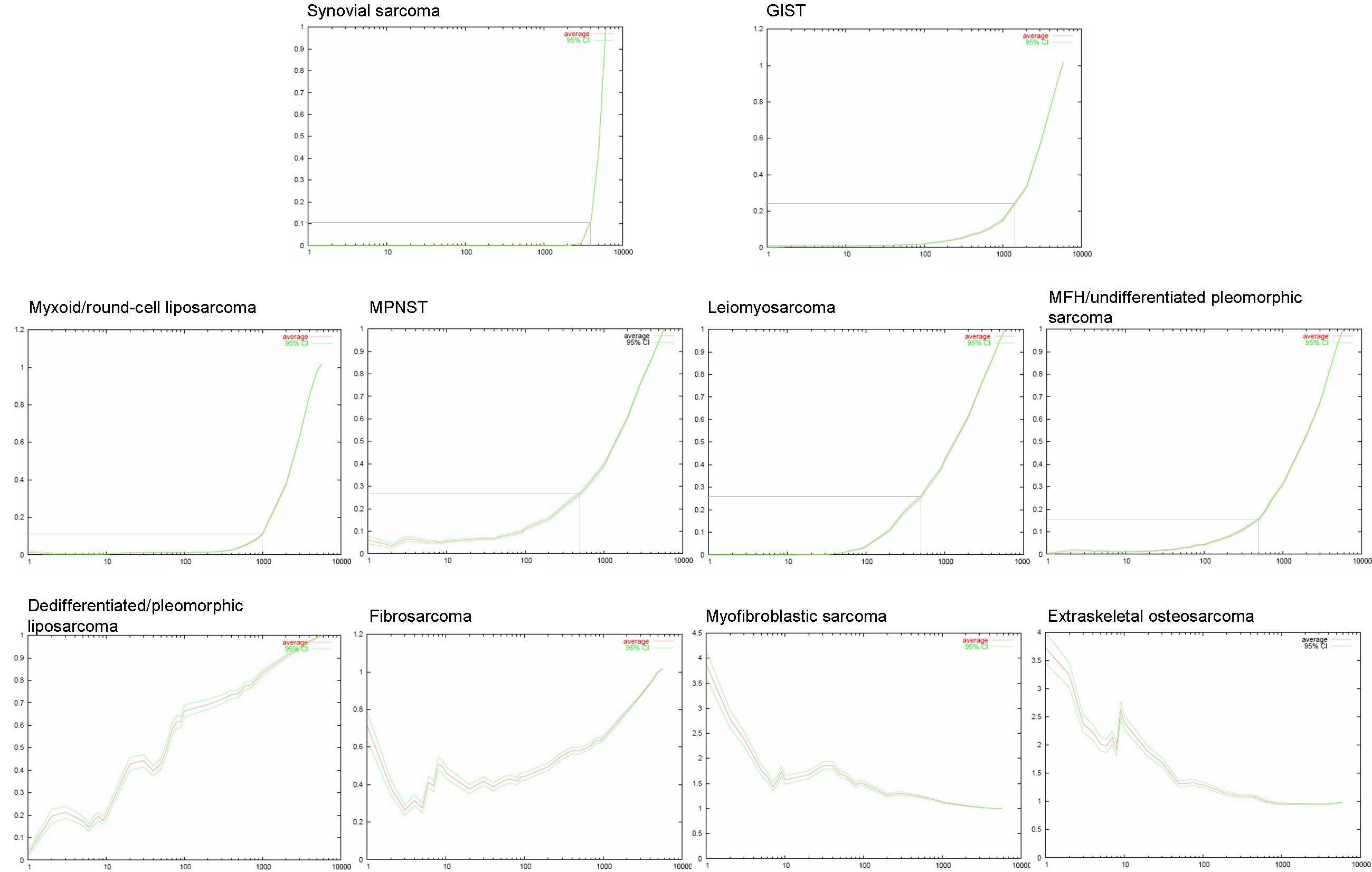

Supplement: Additional file 3 — FDR plots for the diagnostic signatures. FDR plots for the diagnostic signatures with the number of ranked genes plotted along the x-axis and FDR along the y-axis. Random permutation tests with 1000 permutations were performed to assess the discriminating power or robustness of the Golub-score ranked genes. For each rank, the average number of genes in a permutation list above that rank was divided by the number of genes in the true list to get the FDR. [file 1471-2164-8-73-S3.jpeg]

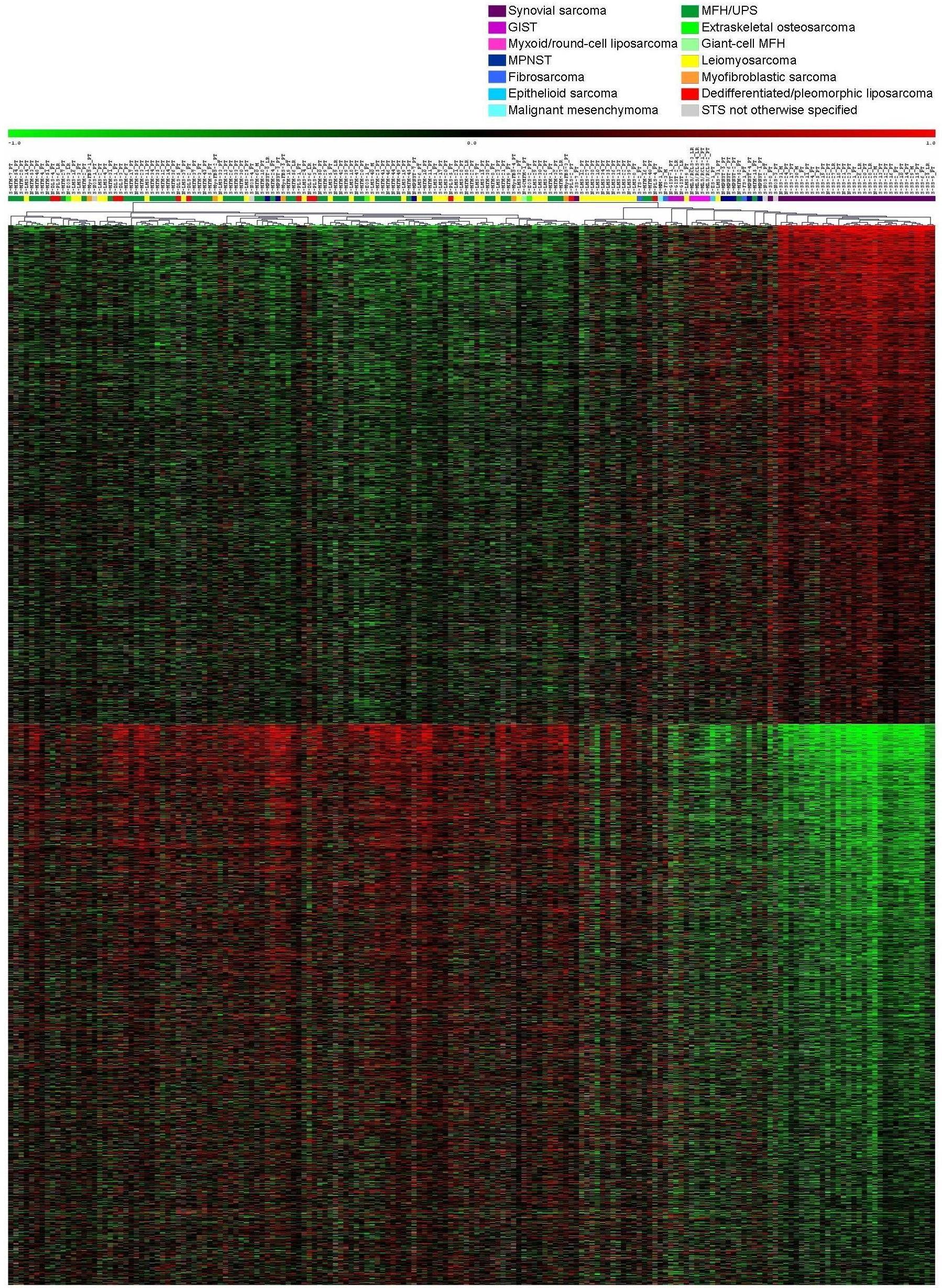

Supplement: Additional file 5 — Supervised clustering based on the SS signature. Supervised clustering of 177 STS samples based on the top 4000 synovial sarcoma discriminating genes. [file 1471-2164-8-73-S5.jpeg]

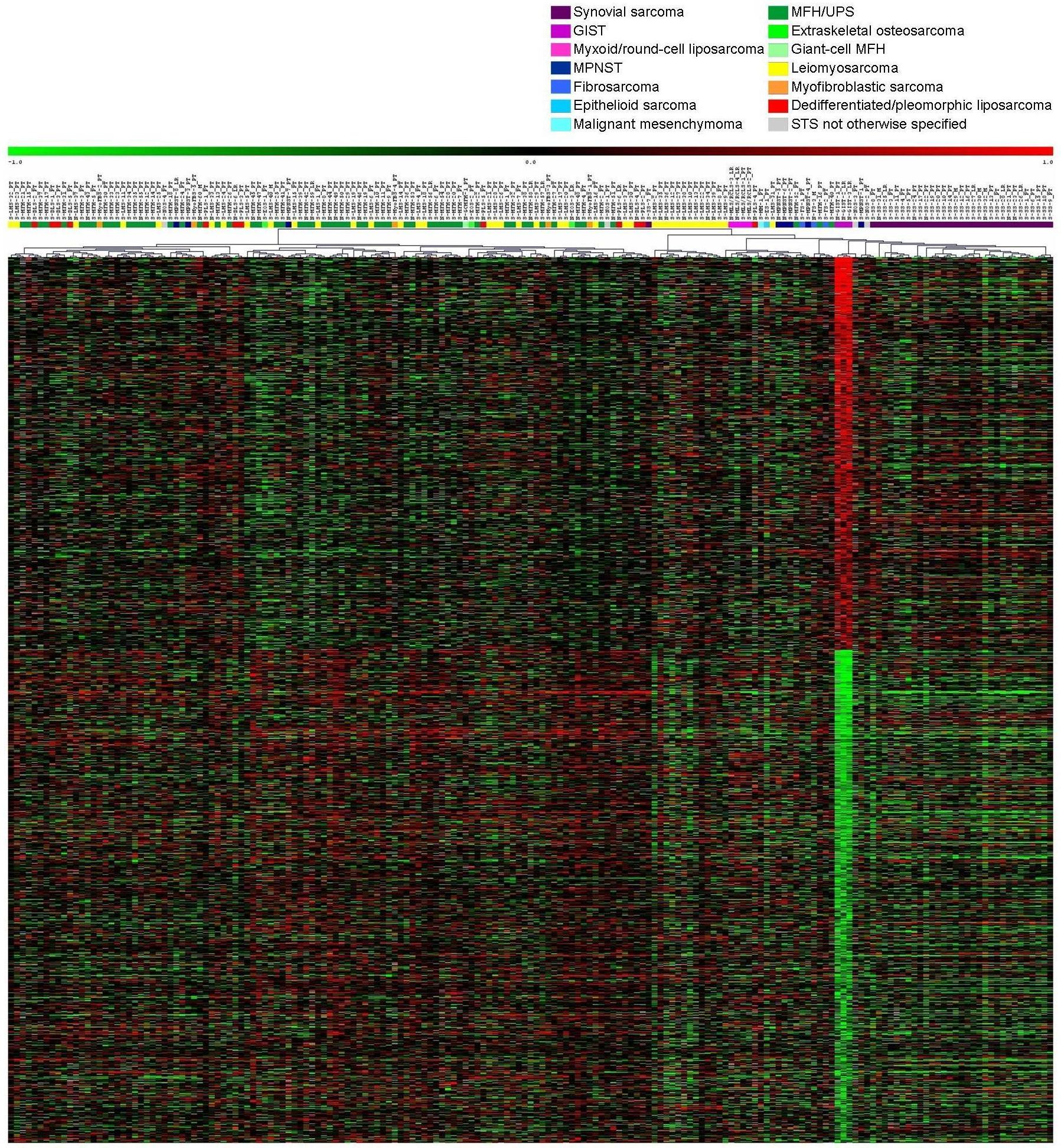

Supplement: Additional file 7 — Supervised clustering based on the GIST signature. Supervised clustering of 177 STS samples based on the top 1500 GIST discriminating genes. [file 1471-2164-8-73-S7.jpeg]

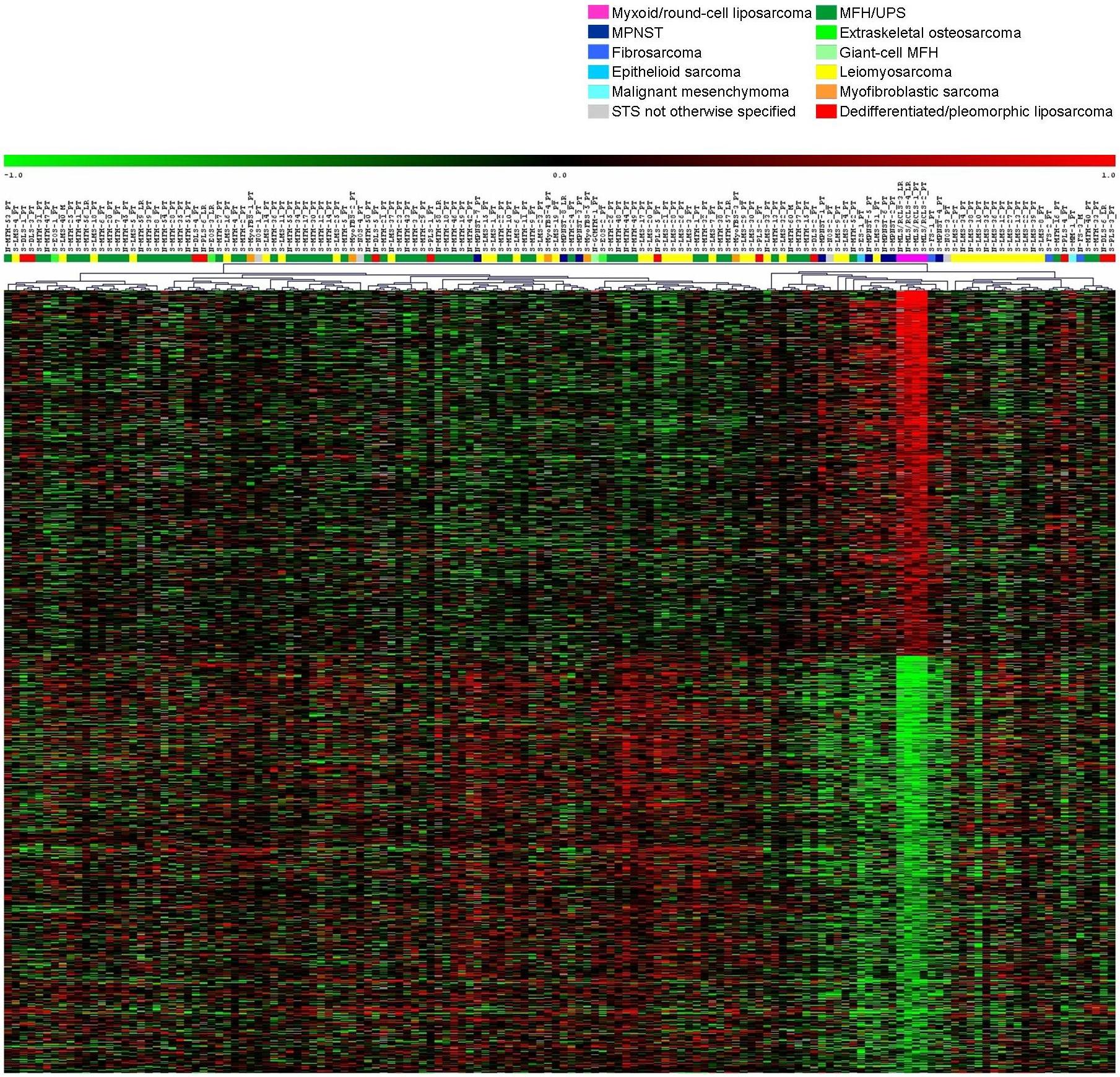

Supplement: Additional file 9 — Supervised clustering based on the myxoid/round-cell liposarcoma signature. Supervised clustering of 142 STS samples based on the top 1000 myxoid/round-cell liposarcoma discriminating genes. [file 1471-2164-8-73-S9.jpeg]

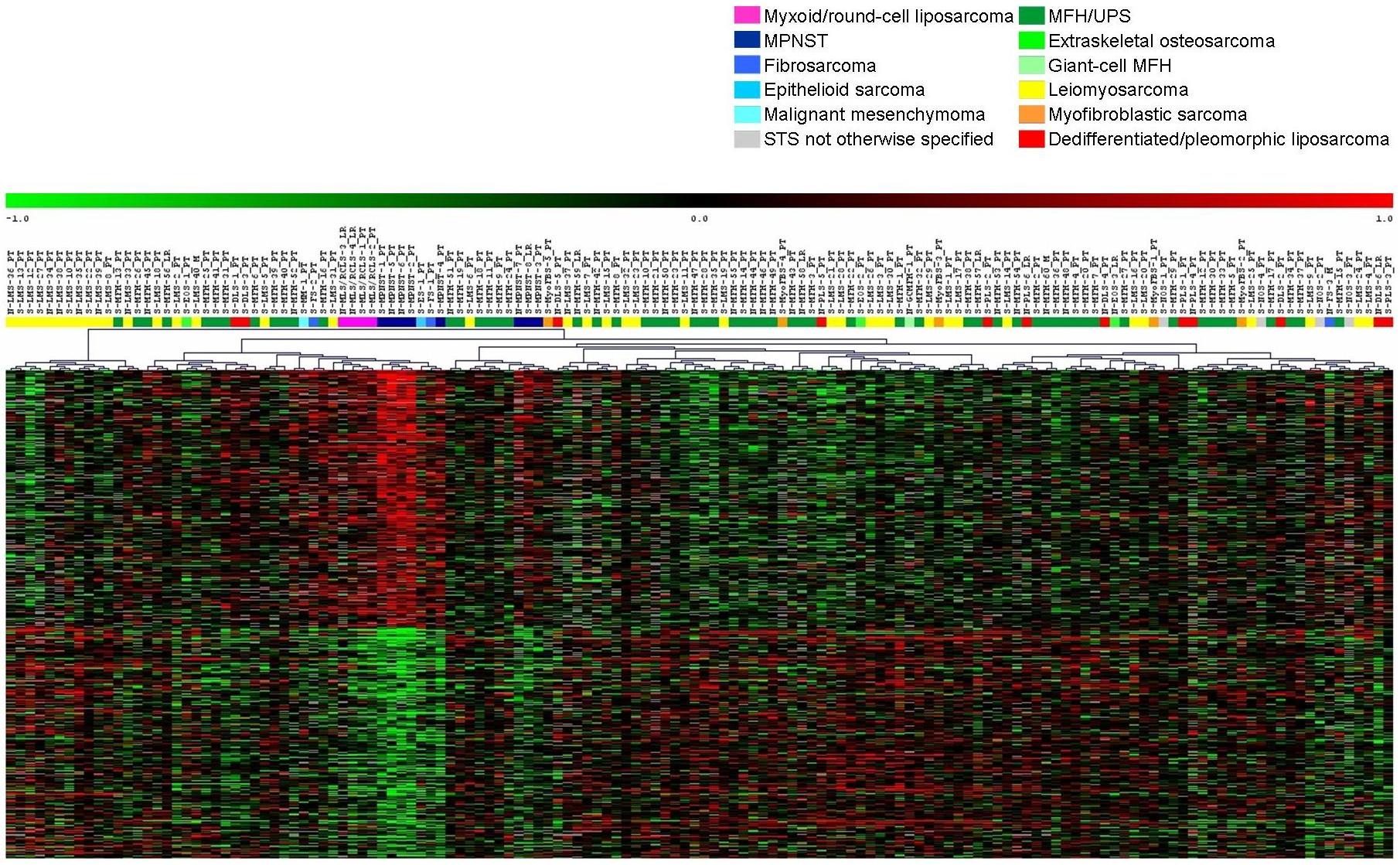

Supplement: Additional file 11 — Supervised clustering based on the MPNST signature. Supervised clustering of 142 STS samples based on the top 500 MPNST discriminating genes. [file 1471-2164-8-73-S11.jpeg]

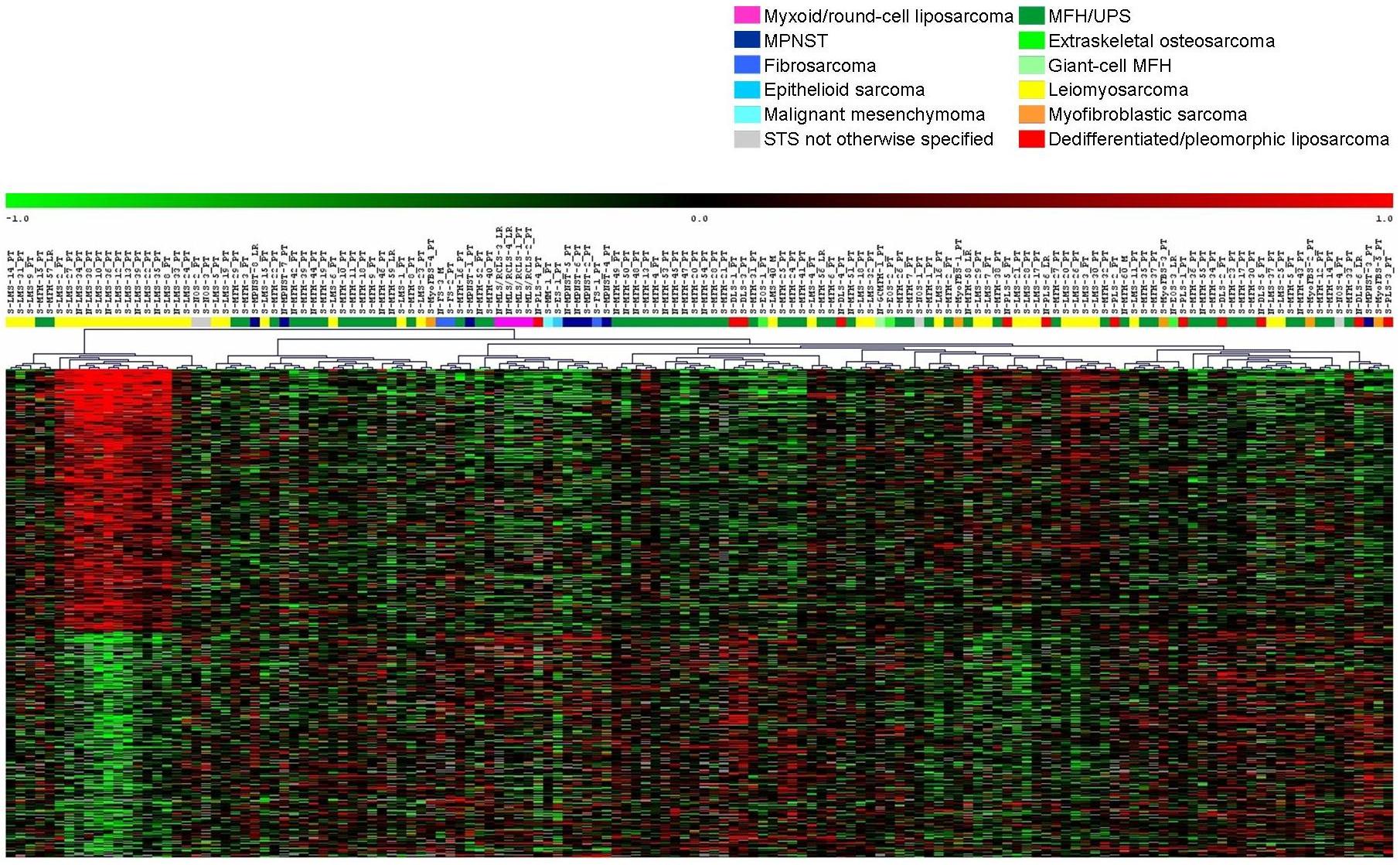

Supplement: Additional file 13 — Supervised clustering based on the leiomyosarcoma signature. Supervised clustering of 142 STS samples based on the top 500 leiomyosarcoma discriminating genes. [file 1471-2164-8-73-S13.jpeg]

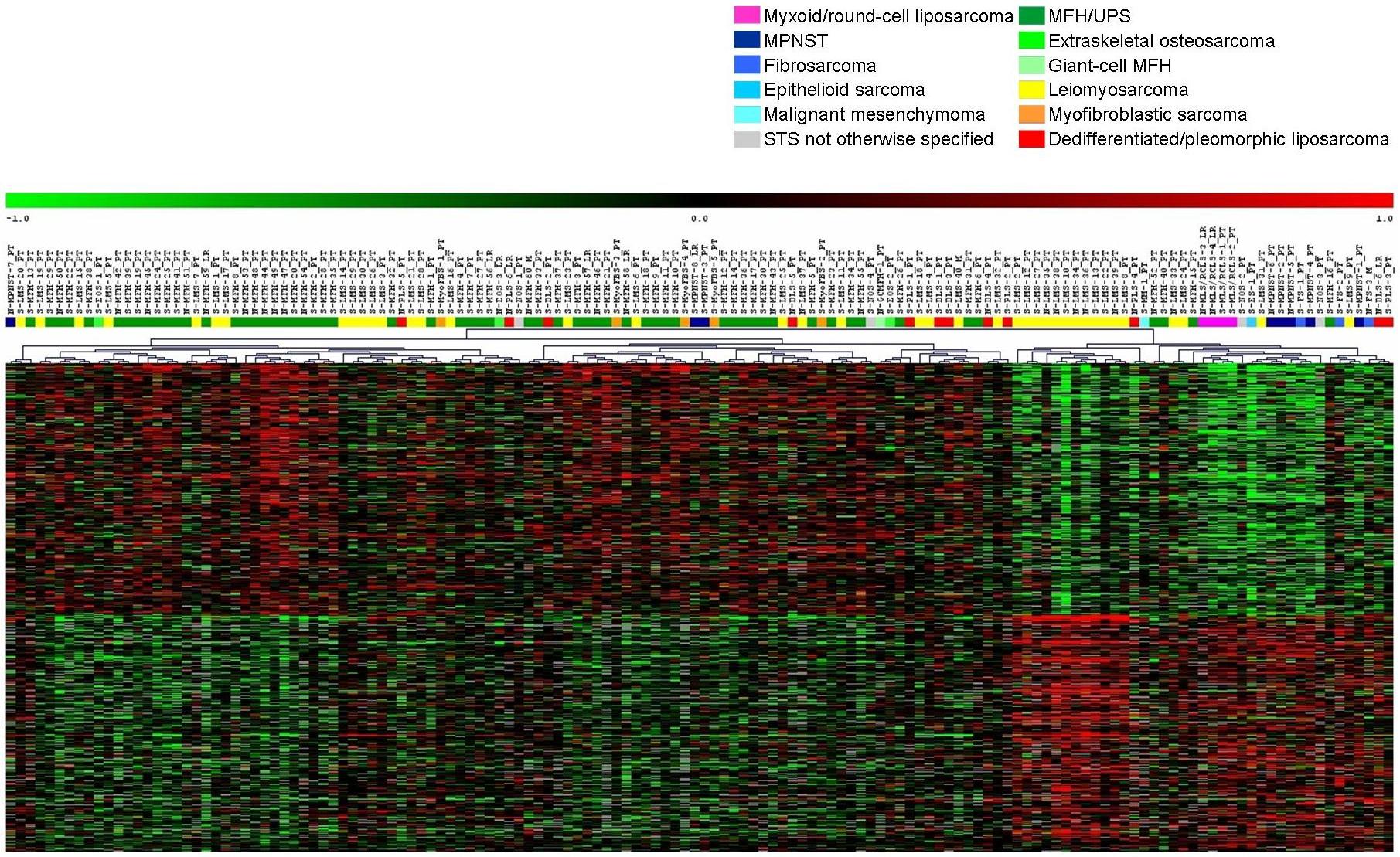

Supplement: Additional file 15 — Supervised clustering based on the MFH/UPS signature. Supervised clustering of 142 STS samples based on the top 500 MFH/UPS discriminating genes. [file 1471-2164-8-73-S15.jpeg]

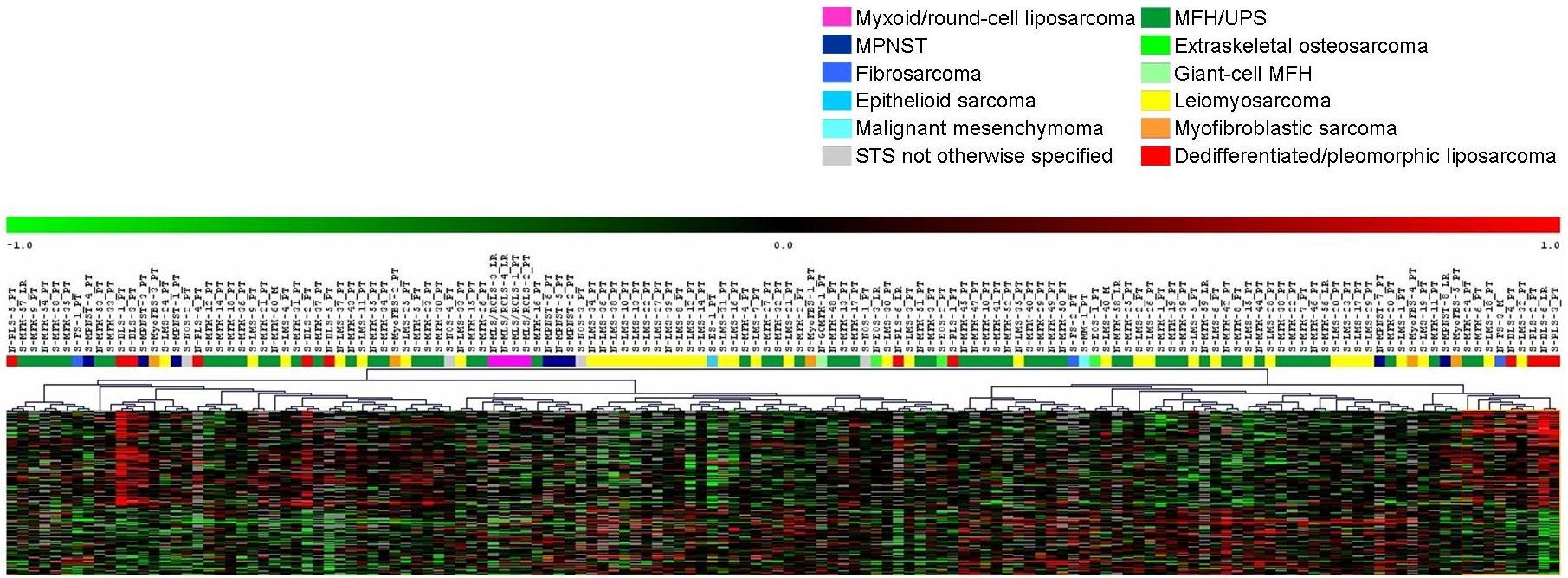

Supplement: Additional file 17 — Supervised clustering based on the dedifferentiated/pleomorphic liposarcoma signature. Supervised clustering of 142 STS samples based on the top 150 dedifferentiated/pleomorphic liposarcoma discriminating genes showing 5/12 dedifferentiated/pleomorphic liposarcomas clustered tightly with 2 MFH/UPS, 2 leiomyosarcomas and 1 fibrosarcoma, all of which possess the CDK4-MDM2 amplification. [file 1471-2164-8-73-S17.jpeg]

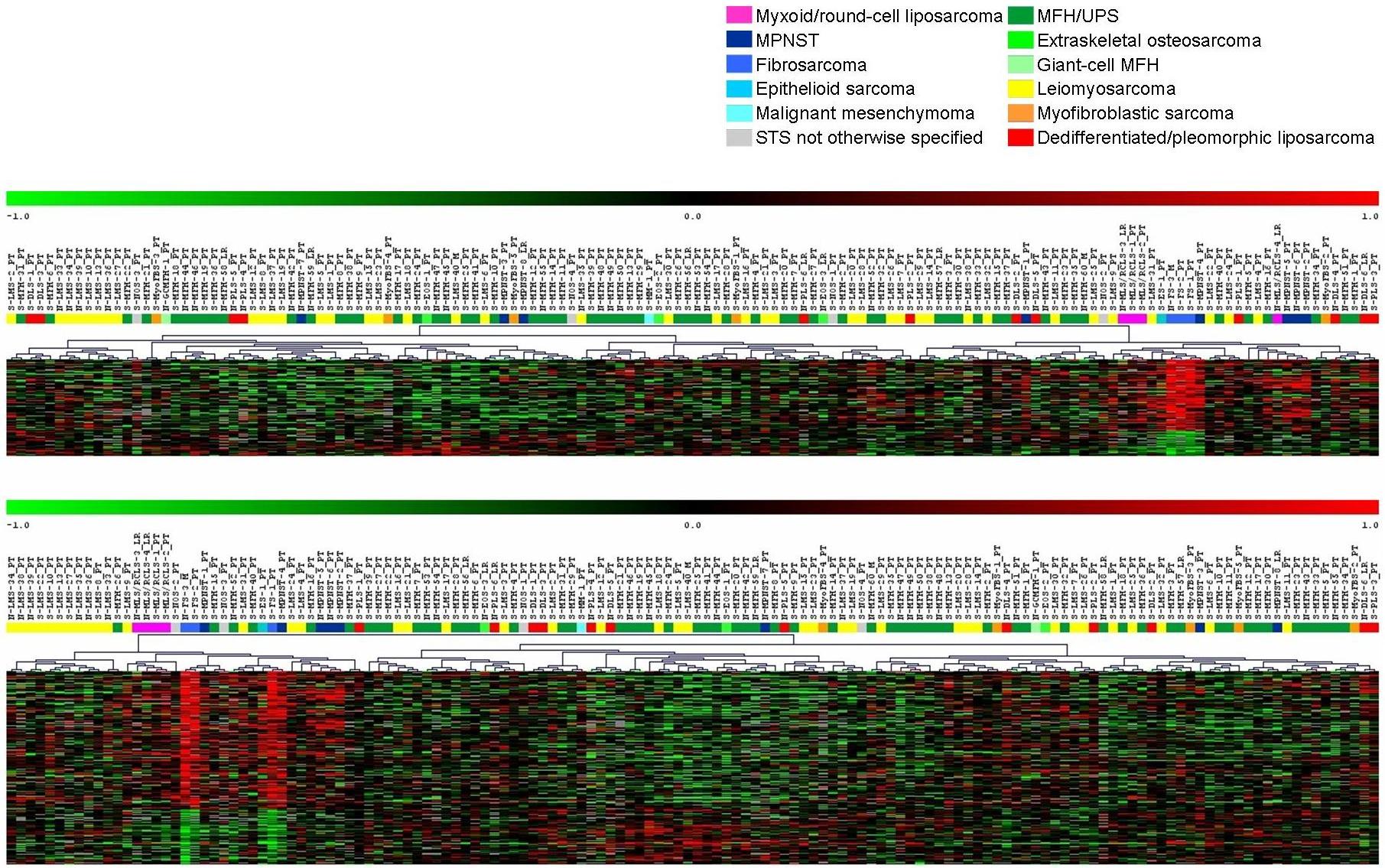

Supplement: Additional file 19 — Supervised clustering based on the fibrosarcoma signature. Supervised clustering of 142 STS samples based on the top 100 (44% FDR) and top 200 (50% FDR) fibrosarcoma discriminating genes. [file 1471-2164-8-73-S19.jpeg]
